# Optical chirality of all dielectric q-BIC metasurface with symmetry breaking

**DOI:** 10.1515/nanoph-2024-0666

**Published:** 2025-03-17

**Authors:** Yujia Sun, Chongjun He, Zilan Deng, Xin Li, Xiaozhi Li, Zhongyuan Zhang, Xiubao Sui, Ning Li, Weiji He, Fangzhou Chen

**Affiliations:** College of Astronautics, Nanjing University of Aeronautics and Astronautics, Nanjing 211106, China; Guangdong Provincial Key Laboratory of Optical Fiber Sensing and Communications, Institute of Photonics Technology, College of Physics & Optoelectronic Engineering, Jinan University, Guangzhou 510632, China; School of Electronic and Optical Engineering, Nanjing University of Science and Technology, Nanjing 210094, China

**Keywords:** optical chirality, circular dichroism, dielectric metasurface, high quality factor

## Abstract

As a two-dimensional material at the nanoscale, optical metasurfaces have excellent and flexible optical field control methods. In particular, the application of the concept of bound states in the continuum (BIC) enables optical metasurfaces to achieve resonance effects with high quality factors (Q factor). In comparison to plasmonic metasurfaces, all dielectric metasurfaces can effectively reduce the Ohmic losses in the structure. In this study, we propose a q-BIC metasurface with a high quality factor (maximum Q factor of 247), which is all dielectric and symmetry-breaking, and investigate the enhancement effect of this structure on optical chirality in the near-infrared band. In the simulation and experiment, the transmission spectra of the structure in the near-infrared band exhibited differences at different light source incidence angles when illuminated with circularly polarised light of varying rotation directions (external chirality). The maximum far-field circular dichroism (CD) achieved was 0.17 in the simulation and 0.038 in the experiment. Subsequently, the near-field chirality enhancement of the structure was investigated, which has the potential to increase the optical chirality of the incident light by up to 22 times. Furthermore, the introduction of a chiral medium to a non-chiral metasurface results in a chiral transfer effect, enabling the achievement of circular dichroism beyond the intrinsic capabilities of the individual substances involved (maximum CD = 0.0055). The high-Q factor of the all-dielectric metasurface paves the way for a plenty of potential applications in optical chiral fields, including chiral sensing, ultra-sensitive analysis of biomaterials and soft matter.

## 1 Introduction

Chirality is used to describe the structural property of a substance and its mirror image, which cannot be overlapped through the application of symmetry operations such as rotation and translation [1]. A group of structures that exhibit this property are referred to as enantiomers [2]. Enantiomers are widely present in the fields of biology and chemistry. For example, the organic compound glyceraldehyde has two configurations: L-type left-handed and D-type right-handed [3], [4]. Despite having the same chemical formula, chiral molecules of different configurations often exhibit disparate chemical properties. A change in the original chirality of biomolecules may result in the loss of functionality or even the production of cytotoxic compounds, which can contribute to the development of various diseases, including short limb deformities, Parkinson’s disease, Alzheimer’s disease, type II diabetes, and Huntington’s disease [5]. Therefore, the accurate and precise detection and characterisation of chiral substances is of great importance in a multitude of fields, such as pharmacology, toxicology, and life sciences [6], [7].

Due to the inherent chiral characteristics of light fields, analytical techniques of optics are particularly well suited to the process of chiral sensing of substances. The interaction of chiral substances with light results in a variety of effects, collectively described as optical chirality. Examples of such effects include circular dichroism and double refraction, which are characterised by intensity and phase differences in the rotational light incident upon the substance [8]. The optical field distribution under the influence of chiral substances is often described by the optical chiral density [9]. The traditional detection technique represented by circular dichroism has a wide range of applications in the fields of protein identification, characterisation and conformational determination [10], [11]. However, traditional detection techniques are associated with limitations, including a low signal-to-noise ratio and the use of complex and expensive equipment. As a small and flexible artificial two-dimensional material, metasurfaces can effectively address many challenges encountered in chiral imaging and sensing in the field of biochemistry [12], [13]. Plasmonic metasurfaces have been first applied to the field of biochiral imaging and substance recognition. For instance, plasmonic metasurfaces can facilitate the recognition of different matter configurations [14], [15], and also enhance the spatial resolution of circular dichroism maps of chiral structures [16].

However, plasmonic metasurfaces exhibit high Ohmic losses, whereas dielectric materials demonstrate low losses and high refractive indices. All dielectric metasurfaces often exhibit distinctive optical characteristics which are different from metallic metasurfaces. These include narrow resonance spectra, remarkable near-field chirality enhancement, and the capacity to support magnetic modes [17]. Presently, some studies have employed all dielectric metasurfaces in the domain of optical chirality enhancement. The strong resonance of all dielectric metasurfaces can induce different configurations of compounds to produce distinct CDs. This process effectively enhances the molecular CD signal in the visible-near infrared (VIS-NIR) spectral range, surpassing the intrinsic molecular CD observed in the ultraviolet (UV) region [18]. Additionally, the coupling between nanoscale mechanical motion and optical chirality in structured semiconductor films has enabled the rapid modulation of linear and circular polarisation [19]. Furthermore, the chirality of the optical field exerts an influence on the generation of substances. The optical chirality near-field enhancement properties of silicon-based metasurfaces can be utilised to guide the probability of different chiral configurations generated during the crystallisation process [20]. However, in these application scenarios, relatively simple periodic arrangements of cylindrical structures are often used, and there is still a lack of discussion and research on other two-dimensional structures.

In order to obtain chiral metasurface structures with sharp resonance waveforms, the concept of bound states in the continuum (BIC) is used as a reference. BIC is a dark state with an infinite quality factor (Q factor), which is caused by destructive interference between multiple radiation channels and therefore cannot couple with external space [21]. The main application is the quasi BIC (q-BIC) with finite Q factor, which can couple with external space and appear in the spectrum. Additionally, it can exhibit sharp resonance waveforms that are not present in general structures [22]. BIC metarsurfaces have found widespread application in fields such as biochemical sensing, nonlinear optics, and wireless optical communications, owing to their exceptional performance [23], [24], [25]. Symmetry-protected BICs are a common mode in all kinds of BIC designs, in which the C2 symmetry of a structure can be broken by changing the structural parameters such as the angle, length, height or position of the structure, and the ideal BIC can be transformed into a q-BIC [26], [27], [28], [29]. Based on this state, the chirality of the structure can be readily designed.

In this paper, a novel all-medium metasurface with an asymmetric circular ring as the periodic structure is proposed. The breaking of the symmetry results in the conversion of the BIC state to the q-BIC state, and the attainment of a sharp resonance waveform (with a *Q* value of up to 247). The introduction of external chirality is achieved through the manipulation of the incidence angle of the light source, and the simulation results demonstrate the achievement of far-field circular dichroism with a maximum value of 0.17 (experimental maximum CD = 0.038). Furthermore, the structure has been demonstrated to enhance the optical chiral field in the near field. The findings of the simulations indicate that the all-medium structure has the capacity to enhance the optical chirality of the incident light by up to 22 times, while also facilitating the chiral transfer of the chiral matter, thereby achieving a CD of up to 7.86 times the magnitude of the matter itself. This study comprehensively explores the optical chiral performance of the all-dielectric q-BIC metasurface under various conditions relevant to optical chiral applications, thus providing a valuable reference point for a wide range of related areas.

## 2 Design of all dielectric metasurface and optical chirality analysis

### 2.1 Q-BIC metasurface design and analysis

**Figure 1:**
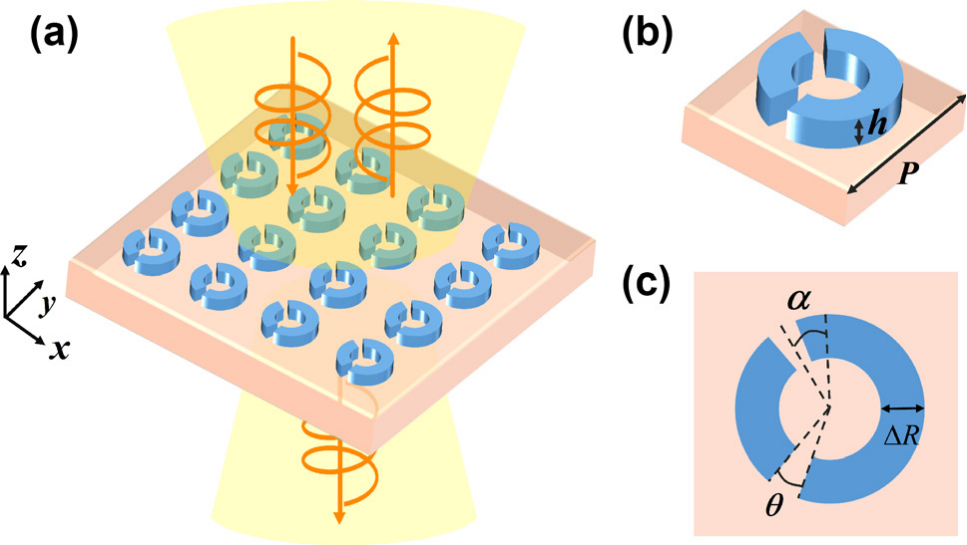
All dielectric q-BIC metasurface. (a) Schematic diagram of all dielectric q-BIC metasurface structure. (b) Unit side view. (c) Unit top view.

In order to obtain metasurfaces with high optical chirality, the principle of bound states in the continuum, which is commonly used in high-Q metasurface design, is applied. The concept of BIC was first introduced in the field of quantum mechanics and subsequently applied to photonics. BIC refers to an eigenstate in which a wave function can propagate to infinity, while energy exists in a bound state within the continuous region. This state corresponds to a special resonance in the radiation continuous region without energy attenuation. It is a dark state in real space, where the resonance linewidth disappears and the quality factor Q is infinitely large [30]. According to the decoupling method between eigenstates and radiation waves, BICs can be divided into symmetry-protected BIC and accidental BICs [31], [32]. Among these, the symmetric protection BIC, which maintains structural symmetry and decouples from the external environment, can achieve a high-Q q-BIC state by simply breaking the symmetry [33], [34]. In order to observe the BIC mode, we consider a single cell composed of split rings resonators (SRR), which are arranged in a square lattice to form an all-dielectric metasurface, as illustrated in Figure 1. The ring is composed of silicon, which exhibits a high refractive index in the near-infrared band, and the substrate is silicon dioxide. The following structural parameters are to be noted: *P* = 0.85 μm, *h* = 0.2 μm, *R* = 0.3 μm, *r* = 0.15 μm, Δ*R* = *R* − r = 0.15 μm, *θ* = 20°. Specifically, *P* denotes the cell size of the periodic structure, *h* denotes the height of the ring within the cell, *R* denotes the outer diameter of the ring, *r* denotes the inner diameter of the ring, *θ* denotes the angular difference between the two partial rings, and *α* denotes the asymmetry angle. In order to eliminate the interference of the substrate on the structural resonance analysis, the focus was initially placed on the resonance characteristics of the SRR. The resonance characteristics of an SRR element were analysed using the finite difference time domain solver, Lumerical FDTD. In the simulation, periodic boundary conditions were employed in the *x* and *y* directions to simulate the infinite periodic structure, while perfect matching layers were utilised in the *z* direction to simulate infinite space in the real situation. In transmittance analysis, a circularly polarised plane wave with a unit amplitude was directed vertically onto the resonator. The specific simulation settings are presented in Supporting Information S1.

**Figure 2:**
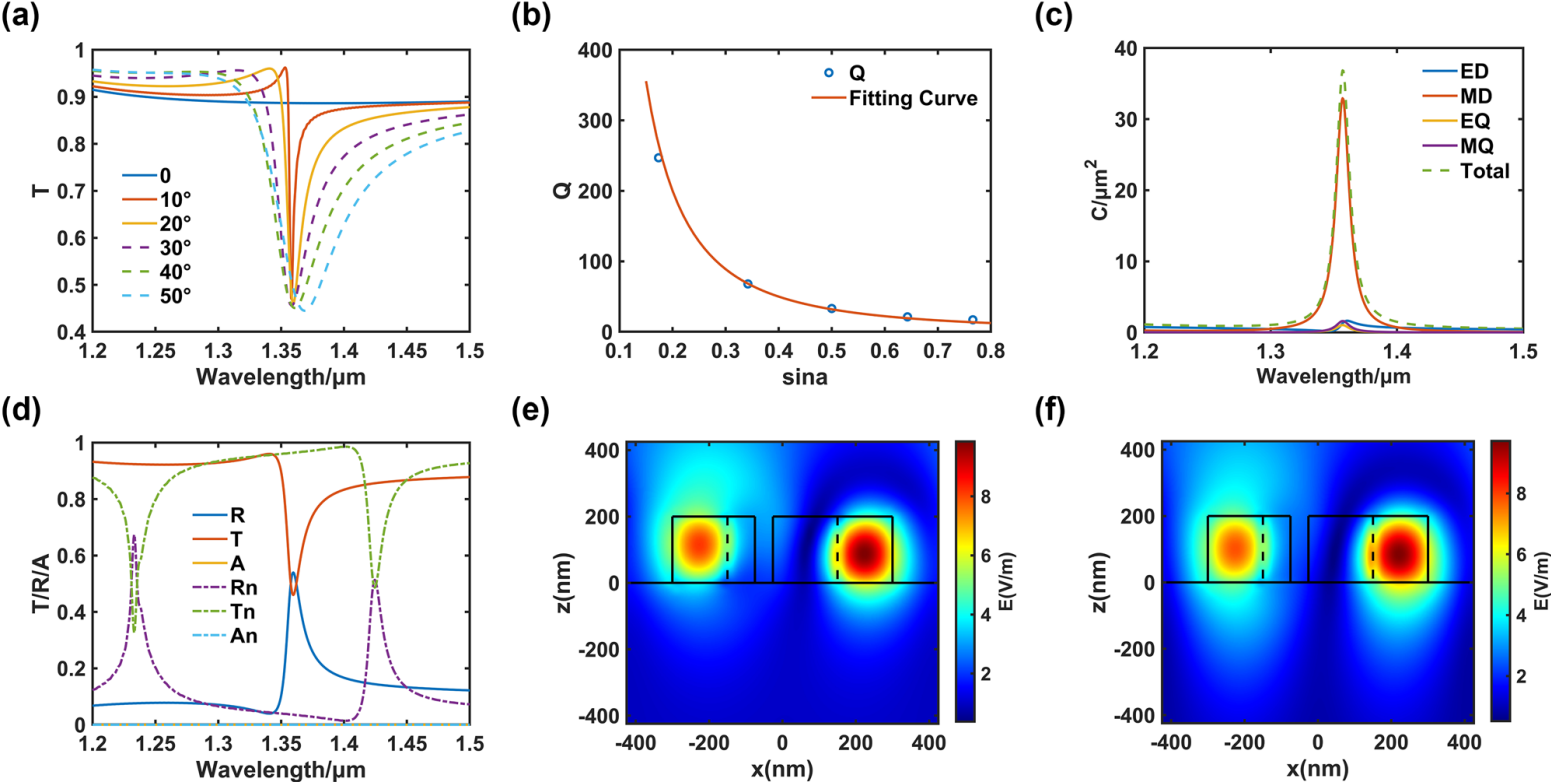
The simulation of QBIC metasurface. (a) Resonance performance of QBIC metasurface. (b) Fitting curve between Q factor and asymmetric parameters. (c) Multipole expansion of the scattering. (d) Transmission reflection absorption spectra without substrate and with substrate. (e) Electric field distribution without substrate. (f) Electric field distribution with substrate.

At normal incidence, the decoupling of BIC states from all scattering channels of the external environment results in the absence of resonance characteristics within the studied band. This is evidenced by the resonance-free wave curve in Figure 2a (blue line). As a consequence of the symmetry mismatch between the mode and the excitation, the resonance disappears from the spectrum at normal incidence. This is due to the fact that the mode is a symmetry-protected BIC, which can only leak out when the symmetry is broken. It is necessary to modify the symmetry of the structure in order to disrupt the C2 symmetry, thereby transforming the unobservable BIC into a leakage state q-BIC that is capable of coupling with the external space. This is illustrated in the multiple resonance wave curves in the region of the 1.36 μm wavelength in Figure 2a. As the asymmetric parameter is reduced, the resonance point of the waveform becomes increasingly sharp. Furthermore, as the asymmetric angle of the structure is increased, the linewidth of the resonant wave is observed to widen significantly. By calculating the Q factors of different q-BICs and coupling them with the asymmetry parameter sin *α*, it was found that the Q factor of q-BICs exhibits an inverse quadratic law with the asymmetry parameter sin *α* of the structure (Figure 2b), which is consistent with the characteristics of symmetric protected BICs [35]. This provides a convenient method for customising the Q factor of the response. Furthermore, the rotation direction of the incident light has no impact on the simulation results. This is evidenced by the fact that, under vertical incidence of the light source, the transmittance of left and right circularly polarised light (LCP, RCP) passing through the structure is identical, indicating that the structure is unable to distinguish the rotation direction of the incident light in this scenario.

The following section will address the electromagnetic mode of the structure. Multipole expansion is a prevalent analytical technique in nano electromagnetics, frequently employed to investigate electromagnetic modes in nanostructures. This approach replaces the intrinsic scattering effect of the structure by superimposing the modes of multipoles. The total scattering cross-section is expressed as the sum of the contributions of distinct multipole moments, as shown in the following formula [36]:

$$\begin{array}{cc} C_{\text{Total}} & =C_{ED}+C_{MD}+C_{EQ}+C_{MQ}+\cdots \\ & =\frac{k^{4}}{6\pi \varepsilon_{0}^{2}{\left|E_{\text{inc}}\right|}^{2}}\left[\underset{a}{\sum }\left({\left|p_{a}\right|}^{2}+\frac{{\left|m_{a}\right|}^{2}}{c}\right)\right.\\ & \;+\frac{1}{120}\underset{a\beta }{\sum }\left({\left|kQ_{a\beta }^{e}\right|}^{2}+{\left|\frac{kQ_{a\beta }^{m}}{c}\right|}^{2}\right)+\cdots \end{array}$$

Among them, *p*_*a*_ and *m*_*a*_ are electric dipole moment ED and magnetic dipole moment MD, respectively. $Q_{a\beta }^{e}$ and $Q_{a\beta }^{m}$ are electric quadrupole EQ and magnetic quadrupole MQ respectively. $\left|E_{\text{inc}}\right|$ is the amplitude of the electric field of the incident plane wave, *k* is the wave number, and *c* is the speed of light. As demonstrated in Figure 2c, the outcome of the multipole expansion process reveals that the primary mode of the structure is the magnetic dipole (orange line), which exhibits a comparable scattering area to that of all the modes combined (green dashed line). The remaining modes, such as the electric dipole (blue line), electric quadrupole (yellow line), and magnetic quadrupole (violet line), collectively account for a significantly smaller area. This outcome can be attributed to the generation of displacement currents in the dielectric medium in response to the incident light, a phenomenon that is more pronounced in the case of magnetic polarization modes. In contrast, metals exhibit a greater tendency to generate electrodipole modes. Furthermore, the exclusion of the ring polariser and other higher-order polarisers from the analysis does not impact the results, as these components can be disregarded in the context of this particular structure. The specific formula for multi-level expansion can be found in Supporting Information S2.

To illustrate the reflection, transmission, and absorption capabilities of an asymmetric structure with an angle *α* of 20°, Figure 2d presents a graphical representation of them (the relationship between the three is *A* = 1 − *R* − *T*). As demonstrated by the solid line in Figure 2d, the results indicate that the asymmetric structure exhibits no absorption near the resonance band (the absorption line represented by the yellow line is 0). The structure is generally deposited on an additional substrate for the ease of practical fabrication, and the commonly used substrate silica in the near-infrared band is used in this design. The simulation results after adding the substrate are shown as the dashed line in Figure 2d, which demonstrates that the position of the trough of the transmission resonance wave (green dashed line) will be red-shifted to a certain extent, moving to wavelengths 1.42 μm; the extreme value of the trough has also been shifted upward, moving by 0.03 or so. The reason for this effect is that the substrate introduces additional losses and dispersion effects to the structure, which leads to modal leakage into the substrate [37]. This phenomenon can be observed by examining the alteration in the electric field cross-section at the resonance wavelength and at position *y* = 0 before and after the addition of the substrate, as illustrated in Figure 2e and f. The region of the figure indicated by *z* < 0 corresponds to the substrate, while the black box represents the SRR structure. More electromagnetic waves are retained in the substrate region in proximity to the SRR within the range of *z* < 0. Figure 2f demonstrates that the q-BIC mode persists mainly within the SRR structure, with only a minor leakage into the substrate. Consequently, the resonant wave valley exhibits only a slight amplitude change. Furthermore, a comparison of the reflection, transmission, and absorption before and after (Figure 2d) reveals that the resonant wave of the substrate emerges at a wavelength of 1.23 μm approximately and does not interfere with the resonant wave of q-BIC.

### 2.2 Simulation and experimental study of metasurface external chirality

**Figure 3:**
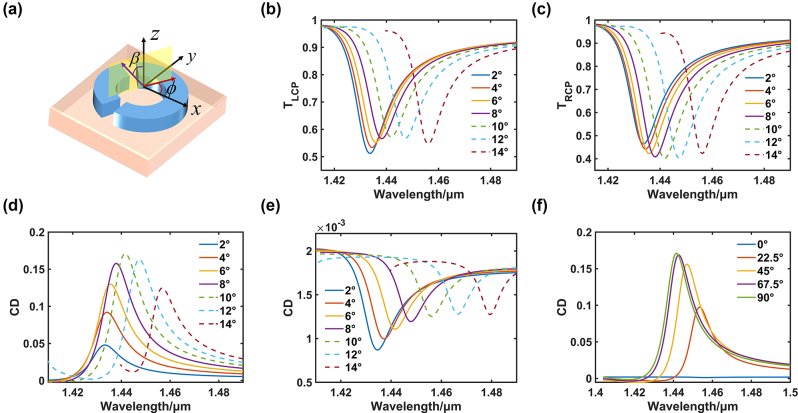
External chirality of the metasurface. (a) Schematic representation of the angle of incidence and azimuth. (b) Transmittance of left-handed circularly polarised waves. (c) Transmittance of right-handed circularly polarised waves. (d) CD with 90° azimuth. (e) CD with 0° azimuth. (f) CD at different azimuth angles.

Based on the previous research on the q-BIC structure, the optical chirality performance of the structure is further discussed. A common chiral property of optical chirality is circular dichroism, which is often used for the detection of chiral substances or for the imaging and transmission of differences in different rotations. There are various expressions for circular dichroism, and in this study a commonly used expression CD = *T*_LCP_ − *T*_RCP_ was used to calculate the difference in transmittance of the SRR structure under circularly polarised waves of different rotation directions in order to explore the potential of the optical chirality performance of the SRR structure. By adjusting the angle of incidence of the light source, external chirality can be introduced and characterised by strong resonance of the SRR structure [38]. As illustrated in Figure 3a, two critical angles are of particular significance when considering the angle of incidence of a light source. One of these angles is the angle *β* (angle of incidence) between the light source and the *z*-axis. The other is the angle *ϕ* (azimuth of incidence) between the light source and the *x*-axis, which is the axis of symmetry of the structure under consideration. Firstly, considering the incidence of light sources with different rotation directions, under the incidence of left-handed circularly polarised waves and the incident azimuth angle *ϕ* = 90°, as the angle *β* of incidence increases, the transmitted wave valley shows a trend of first increasing and then decreasing (Figure 3b); on the contrary, under the incidence of right-handed circularly polarised waves, as the angle *β* of incidence increases, the transmitted wave valley shows a trend of first decreasing and then increasing (Figure 3c). Through the calculation formula of the CD value, it is found that as the incident angle *β* of light increases, the maximum value of CD will show a trend of first increasing and then decreasing, which reflects that the structure achieves maximum external chirality only at a certain angle (*β* = 10°), as we can see from Figure 3d. At this angle, the maximum CD value can reach 0.038. In addition, by varying the azimuth angle of incidence (*β* = 0°), it can be observed that the value is very weak at this angle, indicating that external handedness is only achieved at a certain angle of incidence, as shown in Figure 3e. It can also be observed that the resonant wavelength undergoes a redshift as the angle *β* of incidence increases.

Next, the effect of azimuth on CD is investigated. Keeping the angle of incidence *β* = 10°, the angle of azimuth *ϕ* was varied between 0 and 90°. The transmittance at different angles is first examined, see Supporting Information S3. The circular dichroism is calculated from the transmittance at different rotationally incident waves and the results are shown in Figure 3f. It can be seen that the value of circular dichroism at the peak increases with increasing azimuthal angle. This can be explained by the fact that at an azimuthal angle of 90° the unit structure is symmetric with respect to the *x*-axis vertical to the plane of incidence (the yellow area in Figure 3a), where CD is generally maximum, and, at an azimuthal angle of 0°, the unit structure is not symmetric with respect to the *y*-axis vertical to the plane of incidence, where CD is generally minimum. In addition, it can be seen that in all cases the resonance wavelength is subsequently redshifted as the angle of incidence *ϕ* decreases. The reason for this phenomenon is that the cross sectional area of the unit structure vertical to the incident light increases as the angle of incidence *ϕ* decreases, and the larger resonant structure causes the wavelength at which resonance occurs to increase. It can be seen that the difference in CD values is not very significant for incident angles of 90° and 67.5°, which is due to the fact that the difference in transmittance is also insignificant, as can be seen in Figure S2. Therefore, the advantage of the structure is that it retains significant circular dichroism over a wide range of angles (0 to about 20°).

**Figure 4:**
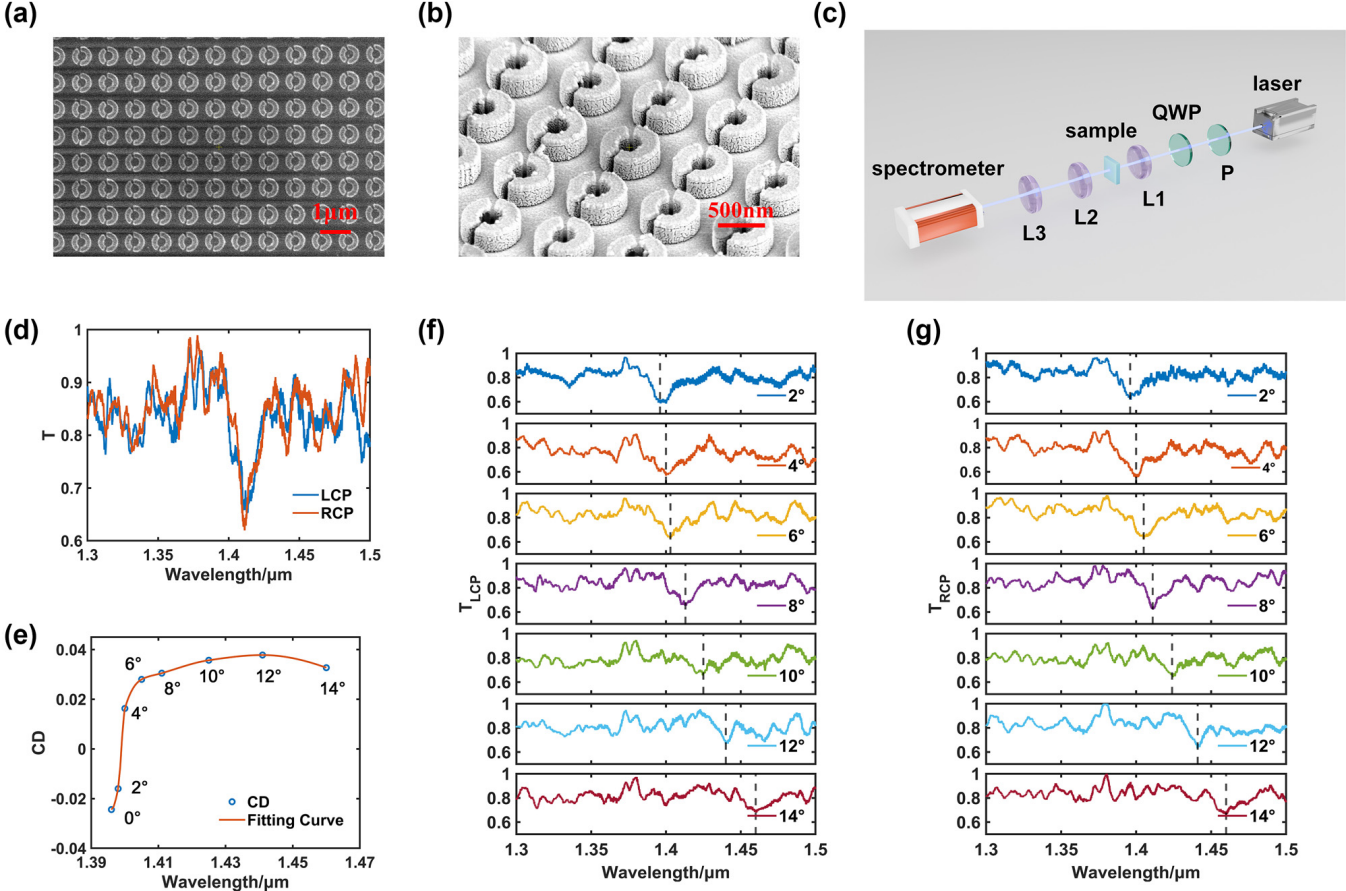
Fabrication and characterisation of the metasurface. (a) and (b) Sample image of the metasurface. (c) Test optical path diagram. (d) Transmittance at 8° incidence. (e) Resonance wavelengths at different angles of incidence and maximum CD. (f) and (g) Transmittance at different angles of incidence under left and right rotation.

Process according to the structure with an asymmetric angle of 20° as the standard. The fabrication of sample follows these steps: Use an optical coating machine to deposit 200 nm Si film on a 2 cm × 2 cm fused silica substrate (pulsed DC power supply, power 300 W, working chamber temperature at room temperature during operation, vacuum degree above 1e-4 Pa). On the prepared Si thin film, AR-P 6200 photoresist was spin coated using a homogenizer. After pre baking treatment, it was exposed using an electron beam lithography system. After exposure, it was developed and residual photoresist was removed using a plasma stripping machine (homogenizer parameter 1,000 r/min, pre and post baking for 5 min, electron beam voltage 125 kV, current 1 nA). Use an electron beam evaporator to deposit 50 nm Cr, and after deposition, heat and peel off with NMP to prepare a Cr hard mask (Cr coating rate of 0.5 A/s, equipment working vacuum degree of 1e-4 Pa or above, room temperature). Use an inductively coupled plasma etching machine to etch the Si film layer, and after etching is complete, use Cr etching solution to remove the Cr mask (etching power 200 W, gas SF6, gas flow rate 800 SCCM, room temperature). The selection of 20° is based on the premise that the quality factor of the structural resonance wave at this asymmetric angle is significantly higher, as evidenced by the simulation presented in Figure 2b. Furthermore, the linewidth of 20 nm is deemed to be optimal for subsequent sample testing, as a linewidth that is too narrow would pose a challenge in terms of capture by the spectrometer. The samples were characterised through the utilisation of focused ion and electron dual-beam electron microscopy. The actual processed metasurface structure is shown in Figure 4a and b. From the figures, it can be seen that the structure has an asymmetric circular ring structure, and the gap characteristics in the ring are also well preserved. The choice of 500 cycles is for ease of subsequent sample analysis, as there is a limit to the range in which the light spot can be focused. In addition, with more cycles, the assumption of infinite cycles in the simulation can be better accommodated. More detailed dimensions of the structure can be found in Supporting Information S4.

By constructing a test optical system (as shown in Figure 4c), the transmittance of the metasurface structure was tested at different incident angles (with the incident azimuth angle *ϕ* = 90°). Using a broadband polarizer (P in Figure 4c) and a quarter wave plate (QWP in Figure 4c) cascaded in a supercontinuum laser to generate circularly polarized incident light, the rotation direction of circularly polarized light is controlled by the rotation of the quarter wave plate. Focus the incident light onto the sample using a 50*×* lens (L1 in Figure 4c), and during the measurement, the sample is mounted on a rotating table to change the incident angle of the light. Subsequently, use a 50*×* lens (L2 in Figure 4c) to convert the light passing through the sample into parallel light. Finally, the light was coupled into the fiber using a 35*×* lens (L3 in Figure 4c) and the transmittance was measured using an infrared spectrometer.

Taking the angle *β* = 8° of incidence as an example, Figure 4d shows the spectra of different transmittances at different types of circularly polarized waves. It can be seen that there is a resonance valley at 1.41 μm and there is a certain difference in the valley values of the two resonance valleys, indicating that external chirality can be introduced into the spectrum of the structure under the angle of incidence of the light source. In addition, it has been observed that the fluctuations in the resonance bands do not conceal the properties of the resonance, and the fluctuations in the other bands are more significant. Consequently, when calculating the CD value, it is preferred to take the value of the valley of the resonance wave in the transmitted wave, where the fluctuations have a lesser effect. If the values of other bands are taken, the fluctuations may result in the appearance of false circular dichroism. By calculation, the positions and CD of the resonance spectrum valleys at different angles of incidence were obtained, as shown in Figure 4e. It can be seen that the position of the resonance wave is gradually redshifted as the angle of incidence increases, which is consistent with the simulation results. In addition, it can be seen that there is a sign reversal between *β* = 2° and *β* = 4° in CD, which may be due to the slight angular deviation of the sample relative to the vertical direction during placement. This phenomenon is also reflected in the delay of the maximum CD, according to the simulation, the maximum CD appears at *β* = 8°, and the test results show that the maximum value appeared near *β* = 10° (maximum CD is 0.038), with a corresponding delay, indicating that *β* = 0° was actually between 2° and 4°. In addition, when the left-hand circularly polarised wave is irradiated on the structure, the minimum value of the valley increases with the increase of the incident angle (Figure 4f). When the right-hand circularly polarised wave is irradiated on the structure, the minimum value of the valley remains within a certain range with the increase of the incident angle (Figure 4g). These are consistent with the trend of simulation. However, compared to simulation, resonance wave valleys allow more light to pass through the structure because the processed structure is not infinitely large. When the light source is obliquely incident, the same effect as infinite period cannot be achieved.

### 2.3 Simulation research on near-field chirality and chirality transfer within metasurface

In addition to introducing external chirality by changing the angle of incidence of the light source, this periodic array also has the effect of enhancing optical chirality in the chiral near-field. The metasurface can effectively enhance the interaction between light and matter. To measure the ability of the metasurface to enhance the interaction between chiral light and matter in the near-field, the optical chirality density is commonly used to characterise it [39]:

C=−ω2c2ImE⃗∗∗H⃗

**Figure 5:**
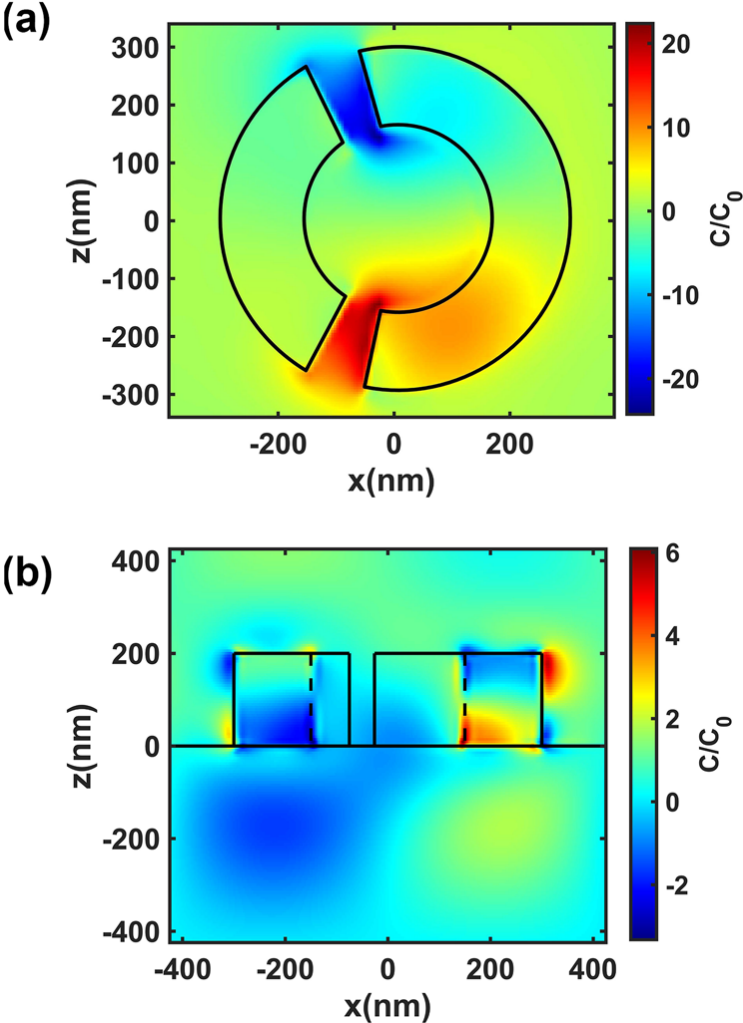
Near-field chirality of metasurfaces. (a) Top view and (b) side view of optical chirality density distribution. (c) The chiral transfer effect of chiral substances.

Among them, *ω* represents angular frequency, *c* represents the speed of light in vacuum, $\vec{E}$ and $\vec{H}$ represents electric and magnetic fields, * represents conjugation. *C* is not zero only when the incident light is circularly polarised, because the condition for the existence of non-zero optical chirality is that the electric and magnetic fields must have parallel components that are out of phase. The previously mentioned structure, which has angular difference *α* = 20°, is selected for the calculation of the enhancement effect of optical chirality at the resonance wave valley 1.42 μm (as shown by green dash line in Figure 2d). This is achieved by utilising the electric *E* and magnetic *H* field values at 1.42 μm that have been derived from the simulation. In theory, the incident light resonates with the structure at this wavelength, and the strongest near-field enhancement exists. Consequently, the optical chiral density of the near-field can be effectively enhanced. The results were normalised by the optical chirality *C*_0_ of circularly polarised light at 1.42 μm, as shown in Figure 5a and b. It can be seen that the enhancement of the metasurface structure can increase the optical chirality field induced by circularly polarised incident light up to 22 times, and the enhancement is mainly concentrated in the narrow slits of the structure, and the chirality field signs in the two slits are different. It should also be noted that the chiral field is an enhancement of the chirality of the incident light by the medium structure. Therefore, for different directions of rotation of the incident light, the two chiral fields have equal magnitudes but opposite values. The enhancement of optical chirality in the near field can have an effect on the formation process of material configurations, such as the probability of the formation of specific configuration substances [20].

The enhancement of optical chirality in the near field can also enhance the optical response of chiral substances, thereby improving the efficiency with which they can be detected. For substances with chiral structures, when they are placed in an array structure with no structural chirality, chiral media will guide the resonance waves of the periodic array to produce response differences under incident waves of disparate rotation directions. The process by which chiral molecules affect the electromagnetic effect between light and non-chiral materials is referred to as chiral transfer [40], [41], [42]. It has been observed that for chiral substances themselves, resonance in the near-infrared band is not exhibited. However, when a chiral substance is applied to a structure that is not chiral, the chiral substance directs the structure to generate chiral currents. The consequence of this is the appearance of circular dichroism in the spectrum, and this effect is the main reason for the generation of chiral transfer [43]. In order to describe the electromagnetic properties of chiral media, the Pasteur constitutive relationship is employed to define chiral media [44]:

$$\begin{array}{c} D=\varepsilon E+i\frac{\kappa }{\text{c}}H\\ B=\mu H-i\frac{\kappa }{\text{c}}E \end{array}$$

**Figure 6:**
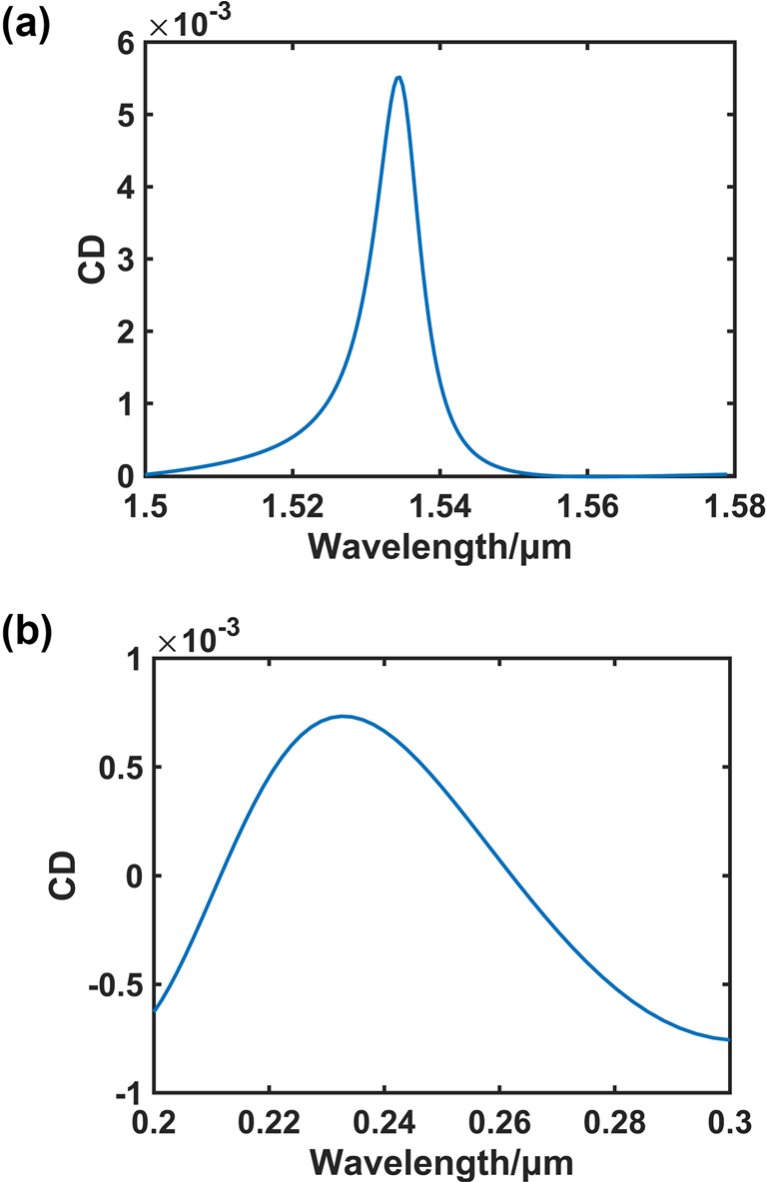
Chirality transfer within metasurface. (a) The chiral transfer effect of chiral substances. (b) Circular dichroism of chiral substances in the ultraviolet band.

Among them, *E* represents the electric field strength, *D* represents the electric displacement vector, *H* represents the magnetic field strength, *B* represents the magnetic induction strength. *ɛ* is the dielectric constant, *μ* is the magnetic permeability, and *κ* is the chiral parameter. The simulation was obtained using the COMSOL finite element solver, and the specific constitutive equation modification is detailed in Supporting Information S5. The thickness of the chiral medium is 200 nm, and in order to simulate the actual situation, it should exist in water [45]. The medium above the metasurface is changed from air to water. In the simulations, a larger chiral parameter *κ* = 0.01*i* is employed to simulate the chiral transfer effect. The simulation is illustrated in Figure 6a, and the CD value of the transmittance peaks around 0.0055. The resonance wavelength is red-shifted in the presence of water, as compared to the resonance wavelength of the green dashed line presented in Figure 2d. In order to demonstrate the enhancement effect of the metasurface, the simulation results of the chiral substance in the UV band are also provided. In addition, the SRR periodic structure is removed from the simulation setup, but the substrate is retained. The result is displayed in Figure 6b, with a maximum CD peak of 7*10–4, in comparison to the circular dichroism enhancement of the metasurface structure by a factor of 7.86. As demonstrated in Figure 6b, the circular dichroism signal of matter exhibits a lower magnitude in the UV band, and the UV band has been observed to impose greater demands on detection equipment. Consequently, there is a particular necessity for signal enhancement and transfer to the visible and infrared bands. Furthermore, it is important to note that for the detection of chiral substances, metasurfaces with chiral optical effects (i.e. metasurfaces have chiral structures) are typically not employed as they can mask the optical chirality of the substance itself. Consequently, metasurfaces that do not possess intrinsic chirality offer a promising approach for chiral substance detection by utilising their chiral transfer properties.

## 3 Conclusions

In this study, we proposed an all-dielectric symmetry-breaking q-BIC metasurface, whose structural design was based on the principle of symmetry-protecting BIC. By breaking the symmetry of the structure, q-BIC resonance waves with sharp waveforms in the near-infrared band were obtained. Furthermore, the structure could be designed to generate resonance waveforms with different shapes by adjusting the asymmetry angle. Following a detailed examination of the modes, it was determined that the resonance was predominantly characterised by magnetic dipole mode. Subsequently, the optical chirality of the structure was subjected to comprehensive analysis with regard to its response to circularly polarised light, employing both simulation and experimental techniques. By modifying the angle of incidence of the circularly polarised light, an external chirality was introduced into the resonance waveform. The results demonstrated that the maximum external chirality was achieved at 10° (simulated maximum CD = 0.17), and the experimental findings were in accordance with the simulation (experimental maximum CD = 0.038). Furthermore, an investigation was conducted into the near-field of the structure, which was observed to enhance near-field chirality when the light source was vertically incident, increasing the optical chirality of the incident light by a factor of 22. Subsequently, the detection performance of structures for chiral substances was investigated, resulting in the achievement of circular dichroism that exceeded that of the substance itself (maximum CD = 0.0055). The findings of our research demonstrate the effectiveness of a q-BIC metasurface structure for different chirality properties. This structure has potential applications in a number of chiral fields, including chiral sensing, chiral substance catalytic crystallisation and chiral substance ultra-sensitive analysis.

## Supplementary Material

Supplementary Material Details

## Supporting Information

Simulation setup; Multilevel decomposition expressions; Sample structure parameter measurements; Simulation built-in equation modifications.
