# Supplementary material for: Optical chirality of all dielectric q-BIC metasurface with symmetry breaking
Source: Nanophotonics. 2025 Mar 17;14(5):559–69. doi: 10.1515/nanoph-2024-0666 (PMC11953724; doi:10.1515/nanoph-2024-0666)
Supplement: Supplementary file 1 — Supplementary Material Details [file j_nanoph-2024-0666_suppl_001.docx]

Supporting Information

**Optical Chirality of All Dielectric Q-BIC Metasurface with Symmetry Breaking**

Yujia Sun^1^, Chongjun He^1^, Zilan Deng^2^, Xin Li^2^, Xiaozhi Li^1^, Zhongyuan Zhang^1^, Xiubao Sui^3^, Ning Li^3^, Weiji He^3^ and Fangzhou Chen^1^*

1 College of Astronautics, Nanjing University of Aeronautics and Astronautics, Nanjing 211106, China.

2 Guangdong Provincial Key Laboratory of Optical Fiber Sensing and Communications, Institute of Photonics Technology, College of Physics & Optoelectronic Engineering, Jinan University, Guangzhou 510632, China

3 School of Electronic and Optical Engineering, Nanjing University Of Science And Technology, Nanjing 210094, China.

Email: [forsterchen@hotmail.com](mailto:forsterchen@hotmail.com)

Section 1. Simulation settings


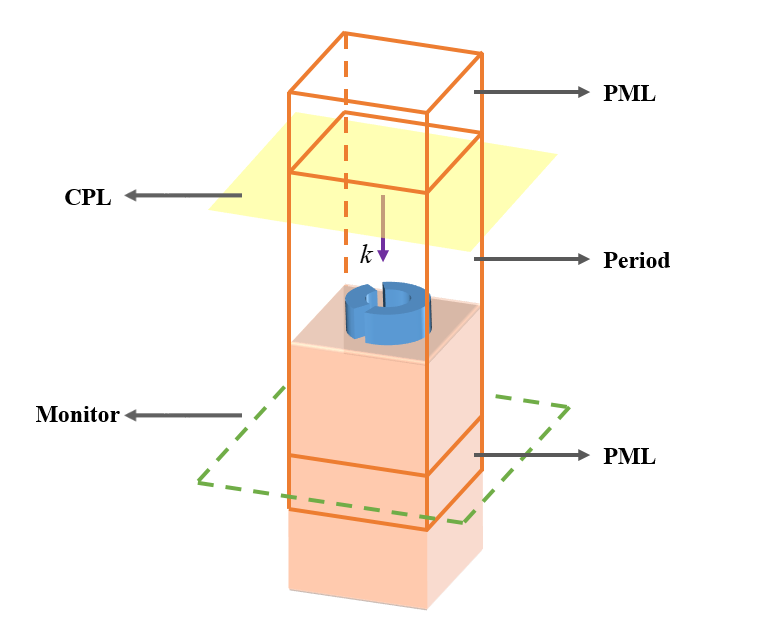


Figure S1 Schematic diagram of simulation settings

The resonance characterisation of a cell of the SRR was conducted utilising the Lumerical FDTD solver. Within the simulation, periodic boundary conditions were applied in the $\text{x}$ and $y$ directions to emulate an infinite periodic structure, while a perfectly matched layer was employed in the direction $z$ to simulate the infinite space of the actual scenario. The substrate thickness (500 µm) is significantly greater in comparison to the periodic structure, and to simulate this effect, PML in the opposite direction of the light source is placed in the substrate. The transmittance monitor is used to observe the transmission of electromagnetic wave energy and is placed in a position above the boundary in the substrate. The transmittance analysis involves the use of two linearly polarized waves with perpendicular polarization directions that are superimposed (with a phase difference set to $\pm\pi/2$) to simulate circularly polarized light (CPL). In addition, left- and right-handed circularly polarized plane waves with unit amplitude are illuminated perpendicularly from the direction $z$ to the resonator.

Section 2. Multipole expansion

Accurate multipole moments are effective for particles of any size and shape. The first four expressions of multipole moment are given below, which are: electric dipole moment ($ED$i.e. $p_{a}$), magnetic dipole moment ($MD$i.e. $m_{a}$), electric quadrupole moment ($EQ$i.e. $Q_{a\beta}^{e}$), and magnetic quadrupole moment ($MQ$i.e. $Q_{a\beta}^{m}$).

$$\text{ED}: p_{\alpha}=-\frac{1}{i\omega}\left\{\int d^{3}\mathbf{r}J_{\alpha}^{\omega}j_{0}\left(kr\right)+\frac{k^{2}}{2}\int d^{3}\mathbf{r}\left[3\left(\mathbf{r}\cdot\mathbf{J}_{\omega}\right)r_{\alpha}-r^{2}J_{\alpha}^{\omega}\right]\frac{j_{2}\left(kr\right)}{\left(kr\right)^{2}}\right\}$$

$$\text{MD}: m_{\alpha}=\frac{3}{2}\int d^{3}\mathbf{r}(\mathbf{r}\times\mathbf{J}_{\omega})_{\alpha}\frac{j_{1}(kr)}{kr}$$

$$\text{EQ}: \begin{matrix} Q_{\alpha\beta}^{\alpha}=-\frac{3}{i\omega}\text{\{}\int d^{3}\mathbf{r}\left[3\left(r_{\beta}J_{\alpha}^{\omega}+r_{\alpha}J_{\beta}^{\omega}\right)-2\left(\mathbf{r}\cdot\mathbf{J}_{\omega}\right)\delta_{\alpha\beta}\right]\frac{j_{1}\left(kr\right)}{kr}\text{]} \\ +2k^{2}\int d^{3}\mathbf{r}\left[Sr_{\alpha}r_{\beta}\left(\mathbf{r}\cdot\mathbf{J}_{\omega}\right)-\left(r_{\alpha}J_{\beta}+r_{\beta}J_{\alpha}\right)r^{2}-r^{2}\left(\mathbf{r}\cdot\mathbf{J}_{\omega}\right)\delta_{\alpha\beta}\right]\frac{j_{3}\left(kr\right)}{\left(kr\right)^{3}} \end{matrix}$$

$$\text{MQ}: Q_{\alpha\beta}^{m}=15\int d^{3}\mathbf{r}\left\{r_{\alpha}(\mathbf{r}\times\mathbf{J}_{\omega})_{\beta}+r_{\beta}(\mathbf{r}\times\mathbf{J}_{\omega})_{\alpha}\right\}\frac{j_{2}(kr)}{(kr)^{2}}$$

Section 3. The influence of azimuth angle on transmittance

Keeping the incident angle $\beta=10^{\circ}$ and varying the incident azimuthal angle between 0 and 90°, the transmittance is simulated at different angles and the results are shown in Fig. S2. It can be seen that the position of the resonance is blue-shifted as the angle increases, regardless of whether the incident light is left- or right-rotating. When the incident light is left-rotating, the transmittance at the trough of the resonance becomes larger as the angle increases. If the incident light is right-rotating, the transmittance at the trough of the resonance becomes smaller as the angle increases.


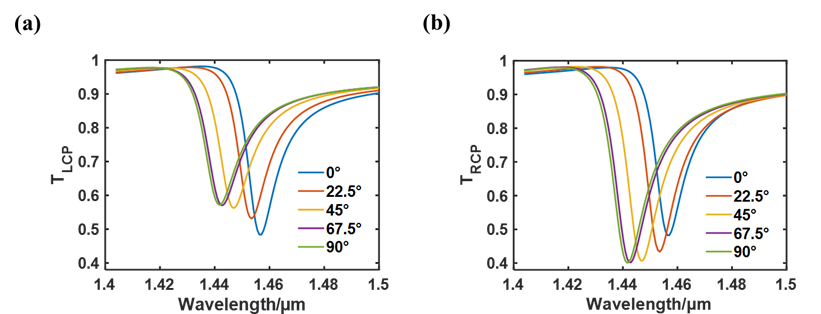


Figure S2 The influence of azimuth angle on transmittance. (a) Transmittance under LCP incidence (b) Transmittance under RCP incidence

Section 4. Processing of metasurface structures


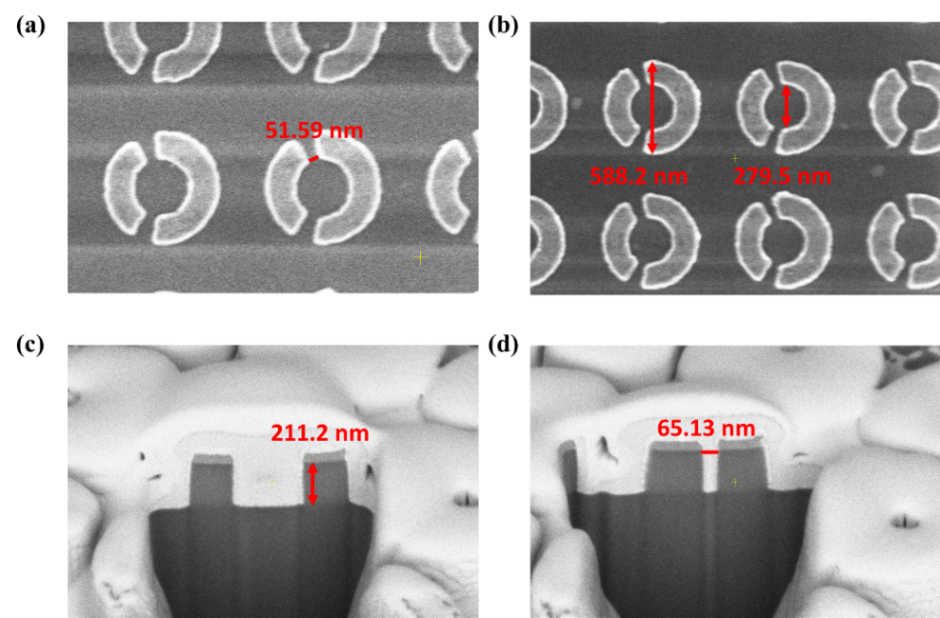


Figure S3 Processing Results. (a) and (b) vertical view. (c) and (d) side view.

The samples were attached to the sample stage with carbon adhesive, passed through the transfer bin into the observation bin, the electron gun diaphragm was opened, the voltage was set to 2kv current 0.1nA, and the secondary electron detector mode was used for characterization observations. From Figure S2, it can be seen that there are slight deviations between the processed structure and the design, but the overall effect is relatively ideal.

Section 5. Modification of constitutive equation

Due to changes in the constitutive equation of chiral media, modifications need to be made to the built-in equations in the simulation. The simulation uses the frequency domain physical field (ewfd) in COMSOL. The specific modifications to the wave equation are as follows, where k is the chiral parameter:

**Electric displacement vector:**

ewfd.Dx=epsilon0_const*ewfd.Ex+ewfd.Px+i*k* ewfd.Hx/c_const

ewfd.Dy=epsilon0_const*ewfd.Ey+ewfd.Py+i*k* ewfd.Hy/c_const

ewfd.Dz=epsilon0_const*ewfd.Ez+ewfd.Pz+i*k* ewfd.Hz/c_const

**Magnetic induction intensity vector:**

ewfd.Bx=-ewfd.curlEx/ewfd.iomega-i*k*ewfd.Ex/c_const

ewfd.By=-ewfd.curlEy/ewfd.iomega-i*k*ewfd.Ey/c_const

ewfd.Bz=-ewfd.curlEz/ewfd.iomega-i*k*ewfd.Ez/c_const

**Magnetic induction intensity time deviation:**

ewfd.dBdtx=-ewfd.curlEx+k*ewfd.omega*ewfd.Ex/c_const

ewfd.dBdty=-ewfd.curlEy+k*ewfd.omega*ewfd.Ey/c_const

ewfd.dBdtz=-ewfd.curlEz+k*ewfd.omega*ewfd.Ez/c_const
